# Effects of Occupational Stress on Blood Lipids, Blood Sugar and Immune Function of Doctors

**Published:** 2019-05

**Authors:** Wenjuan WANG, Hui REN, Qiuye TIAN, Chunling TANG, Wenjuan MENG

**Affiliations:** 1. Department of Human Resources, The First Hospital Affiliated of Harbin Medical University, Harbin, P.R. China; 2. Department of Infection Control, The First Hospital Affiliated of Harbin Medical University, Harbin, P.R. China; 3. Commission for Discipline, The First Hospital Affiliated of Harbin Medical University, Harbin, P.R. China; 4. Mental Health Center, The First Hospital Affiliated of Harbin Medical University, P.R. China; 5. Department of Comprehensive Archives, The First Hospital Affiliated of Harbin Medical University, Harbin, P.R. China

**Keywords:** Occupational stress, Blood lipids, Blood sugar, Immune function

## Abstract

**Background:**

We aimed to investigate the effects of occupational stress on blood lipids, blood glucose and immune function of doctors.

**Methods:**

In 2017, 1291 doctors (565 males, 726 females) in The First Hospital Affiliated with Harbin Medical University (Harbin, China) were enrolled based on the principle of convenience of sampling and cluster sampling. Questionnaires were used to investigate demographic characteristics and occupational stress related factors. Level of glycated hemoglobin was detected by immunoturbidimetric method. Concentration of triglyceride was determined by glycerol phosphate oxidase end point method. Total cholesterol concentration in serum was determined by total cholesterol oxidase end point method. Concentration of serum immunoglobulin was detected by immunoturbidimetry.

**Results:**

Levels of glycated hemoglobin and triglyceride in high tension group were higher than those in the low tension group. Levels of IgG and IgM in high tension group were lower than those in low tension group. The risk of elevated glycated hemoglobin levels in > 50-yr-old age group was higher than that of the =<35-yr-old age group. Those in the high coping strategy group was higher in the low coping strategy group. The risk of elevated total cholesterol levels in drinkers is 1.158 times that of non-drinkers. The risk of IgG concentration reduction in smokers was 0.428 times that of non-smokers. The risk of a decrease in IgA concentration in doctors with good sleep quality is 1.527 times that of those with poor sleep quality.

**Conclusion:**

Occupational stress can lead to increased blood lipids and sugar levels as well suppression of immune function in doctors.

## Introduction

The World Health Organization calls occupational stress a worldwide epidemic. Studies have shown that (1–7), the human body in a state of tension may show a series of physiological, biochemical, endocrine, metabolic and immune function changes. In recent years, domestic and foreign scholars have carried out research on the effects of occupational stress on cardiovascular diseases, metabolic diseases and immune system diseases for typical occupational groups such as police and teachers. However, due to different occupational characteristics, the stress evaluation models have different focuses, leading to differences in research conclusions (1–6).

Doctors are prone to occupational stress. Imbalance between doctors and patients, increasing workload, complex doctor-patient relationships, unfair social assessments, and rapid changes in modern medical models lead to higher level of tension in doctors than in general population as well as teachers, police and employees (7).

In this study, we enrolled doctors of First-Class Hospital as the research subjects, and adopted the more mature pay-reward imbalance questionnaire (ERI) and occupational stress related factors scale (“Coping Strategy Scale”, “Emotional Scale”, “Sleep Disorder Scale”) (8, 9), to explore the impact of occupational stress on doctors’ blood lipids, blood sugar and immune function, as so to provide a theoretical basis to assess their physical and mental health.

## Materials and Methods

### Subjects

A total of 1291 clinical doctors in The First Hospital Affiliated with Harbin Medical University (Harbin, China) were randomly selected for questionnaire sampling and laboratory indicators detections in 2017. None of the subjects had a history of neuro-endocrine and immune system diseases. They had not taken the relevant hormonal drugs in the most recent month and all participants signed informed consent.

This study passed the review of the Ethics Committee of The First Hospital Affiliated with Harbin Medical University.

### Questionnaire survey

To investigate the basic situation of the research subjects and factors related to occupational stress. The basic situation survey includes demographic characteristics such as department, gender, age, current working age, marital status, education level, smoking, drinking, etc., as well as past disease history, family disease history, presence or absence of perennial medication, physical exercise, etc. Occupational stress-related factors mainly include occupational stress, stress relief factors, mood and sleep quality. Effort-reward imbalance (ERI) was used to measure occupational stress levels, including factors such as effort, internal input, and reward. A total of 23 items were designed for the questionnaire. The effort included 6 items; the reward included 11 items, and the internal input included 6 items. Each item was scored using the Likert 4-point assignment method (completely disagree, disagree, agree, and fully agree). The effort/reward ratio is the core evaluation index of occupational stress. The calculation formula is: effort score / (reward score×0.5454), the greater the pay/reward ratio, the higher the degree of occupational stress, with the “effort/reward ratio=1” as the boundary value, the effort/reward ratio>1 is high tension group and effort/reward ratio<=1 is low tension group. “Response Strategy Scale” consisting of 10 items was used to measure stress mitigating factors (response strategies). Numbers 1, 2, 3, 4, 5 and 6 after each item represent: I never use it, I rarely use it, I don’t use it, I use it, I use it frequently and I use it very frequently. The sum of the scores is used as a measure of coping strategies, and the higher the score, the more coping strategies. Positive emotions (5 items) and negative emotions (5 items) were measured by “Emotional Scale”. The positive emotion entry answer “Yes” for 1 point and the negative emotion entry answers “Yes” also for 1 point. Quality of sleep is evaluated using “Sleep Disorder Scale”, which is widely used in the field of domestic occupational stress research. “Sleep Disorder Scale” consists of 14 items, such as sleep habits and sleeping quality of the past month. The cumulative score of each item is recorded as a total score. The higher the score, the more serious the sleep disorder and the worse the sleep quality.

### Determination of biochemical indicators

Fasting venous blood (2 ml) was extracted between 8:30 ∼ 9:30 am. Level of glycated hemoglobin was detected by immunoturbidimetric method. Concentration of triglyceride in serum was determined by glycerol phosphate oxidase end point method. Total cholesterol concentration in serum was determined by total cholesterol oxidase end point method. Concentration of serum immunoglobulin (IgG, IgM, IgA) was detected by immunoturbidimetry. All operations were performed in strict accordance with manufacturer’s instructions.

### Statistical analysis

Database was built in Excel and data was statistically analyzed using SPSS 13.0 software (Chicago, IL, USA). Differences in glycosylated hemoglobin levels, triglycerides, total cholesterol, and IgG, IgM, and IgA concentrations between demographic groups were compared using t-test or analysis of variance (LSD test for comparisons between groups). Demographic characteristics were controlled to perform partial correlation analysis on relationships between occupational stress related factors and glycosylated hemoglobin, triglycerides, total cholesterol, IgG, IgM and IgA concentrations. Demographic characteristics/tension factors, emotions, sleep quality, and coping strategies were used as independent variables (participants were divided into high and low score groups according to the mean values of tension factors, emotions, sleep quality, and coping strategies; variable assignment: high Score group=1, low score group=0). Glycated hemoglobin levels, triglycerides, total cholesterol, and IgG, IgM, and IgA concentrations were divided into high and low level groups to serve as dependent variables (variable assignment: high level group=0, low level group=1). Multivariate unconditional logistic regression analysis was performed. The difference was statistically significant at *P*<0.05.

## Results

### Questionnaire and demographic characteristics

Overall, 1243 questionnaires were retrieved, and the response rate was 96.3%. After removing the 13 questionnaires with indicators missing, there were 1230 valid questionnaires. Department composition: there are 592 physicians, 561 surgeons, 18 emergency doctors, 37 maternal and child doctors, and 22 doctors in the fields of skin, ophthalmology, otolaryngology and other departments. There were 538 males and 692 females. The age was (41.63 ± 9.12) yr old. There were 237 smokers and 241 drinkers. There were 1194 shift workers. There were 1198 doctors with education level equal to or higher than college, 911 cases of married doctors.

### Comparisons of glycated hemoglobin, triglyceride, total cholesterol and IgG, IgM, IgA levels between demographic characteristics groups

Glycosylated hemoglobin level in > 50-yr-old age group was higher than that in the <= 35-year-old age group. Glycosylated hemoglobin level of surgeons and emergency doctors was higher than that of doctors in skin, ophthalmology, otolaryngology and other departments (*P*=0.037, 0.040). The triglyceride level in > 50-yr-old age group was higher than that in the <= 35-yr-old age group (Table 1). Triglyceride levels of married and drinkers were higher than those of the control group (*P*=0.043, 0.041). IgM concentration of emergency doctors was higher than that of doctors in skin, ophthalmology, otolaryngology and other departments (*P*=0.036). IgM concentration in females was higher than that in males, and the difference was statistically significant (*P*=0.042). IgG concentration of smokers was lower than that of non-smokers, and the difference was statistically significant (*P*=0.040). There were no significant differences in glycosylated hemoglobin, triglyceride, total cholesterol, and IgG, IgM, and IgA levels among groups (Table 2).

**Table 1:** Glycated hemoglobin, triglyceride, total cholesterol and IgG, IgM, IgA levels

***Variable***	***Lowest value***	***Highest value***	***Mean value (x̄ ± s)***	***Normal reference value***	***Abnormal rate***
Glycated hemoglobin	3.3%	7.2%	(5.12±0.21)%	3.0%∼6.0%	1.2%
Triglyceride	0.41mmol/L	2.66 mmol/L	(1.52±0.95)mmol/L	0.40 mmol/L∼1.70mmol/L	28.8%
Total cholesterol	2.02mmol/L	6.33 mmol/L	(4.87±0.77)mmol/L	2.30 mmol/L∼5.70mmol/L	29.6%
IgG	6.23g/L	18.12g/L	(11.43±3.08)g/L	8.0 g/L∼17.0 g/L	11.1%
IgM	0.27g/L	3.31g/L	(1.18±0.36)g/L	0.29 g/L∼3.44g/L	0
IgA	0.61g/L	4.22g/L	(2.38±0.59)g/L	0.72 g/L∼4.29g/L	8.6%

**Table 2:** Comparisons of glycated hemoglobin, triglyceride, total cholesterol and IgG, IgM, IgA levels between demographic characteristics groups

***Demographic characteristics***		***Glycated hemoglobin level (%)***	***Triglyceride (mmol/L)***	***Total cholesterol (mmol/L)***	***IgG (g/L)***	***IgM (g/L)***	***IgA (g/L)***
Age (years)	<=35	5.09±0.23	1.49±0.86	4.83±0.72	11.52±3.03	1.19±0.41	2.39±0.55
	35∼50	5.12±0.34	1.51±0.91	4.85±0.78	11.36±3.11	1.20±0.32	2.37±0.59
	>50	5.15±0.29^a^	1.58±0.84^a^	4.85±0.67	11.47±3.05	1.19±0.39	2.40±0.55
Department	Veterinarian	5.12±0.36	1.49±0.77	4.84±0.76	11.42±3.14	1.18±0.34	2.41±0.55
	Surgeon	5.18±0.27^b^	1.51±0.69	4.85±0.74	11.41±3.09	1.19±0.29	2.43±0.57
	Emergency doctor	5.16±0.34^b^	1.50±0.68	4.89±0.88	11.44±3.17	1.23±0.37^b^	2.39±0.61
	Maternal and child doctor	5.13±0.25	1.54±0.84	4.80±0.81	11.39±3.17	1.20±0.35	2.40±0.58
	Skin, ophthalmology, otolaryngology and other departments	5.10±0.28	1.52±0.76	4.84±0.71	11.40±3.12	1.16±0.34	2.42±0.56
Gender	Male	5.11±0.37	1.52±0.76	4.87±0.70	11.46±3.21	1.15±0.38	2.40±0.56
	Female	5.12±0.44	1.51±0.84	4.86±0.69	11.39±3.19	1.21±0.37^c^	2.37±0.61
Smoking	Yes	5.13±0.34	1.53±0.69	4.88±0.74	11.37±3.19 d	1.17±0.41	2.42±0.58
	No	5.11±0.26	1.50±0.88	4.86±0.79	11.47±3.07	1.19±0.47	2.40±0.61
Drinking	Yes	5.13±0.21	1.57±0.62^e^	4.87±0.78	11.40±3.12	1.18±0.44	2.41±0.54
	No	5.12±0.32	1.47±0.85	4.86±0.73	11.42±3.09	1.18±0.46	2.38±0.55
Marital status	Unmarried (or divorced, widowed)	5.12±0.24	1.48±0.78	4.84±0.69	11.44±3.10	1.19±0.48	2.39±0.51
	Married	5.12±0.31	1.56±0.64^f^	4.86±0.75	11.43±3.04	1.16±0.42	2.40±0.55

Notes: Compared with <=35 years old group,

a *P*<0.05; compared with skin, ophthalmology, otolaryngology and other departments,

b *P*<0.05; compared with males,

c *P*<0.05; compared with non-smokers,

d *P*<0.05; compared with non-drinkers,

e *P*<0.05; compared with unmarried (or divorced, widowed) doctors,

f *P*<0.05

### Partial correlation analysis between occupational stress-related factors and glycosylated hemoglobin, triglyceride, total cholesterol and IgG, IgM, IgA levels

Negative emotion was positively correlated with glycosylated hemoglobin concentration (*P*=0.035), and coping strategy was negatively correlated with glycosylated hemoglobin concentration (*P*=0.036).

Internal input was positively correlated with triglyceride concentration (*P*=0.008). Negative emotion and total Cholesterol concentration were negatively correlated (*P*=0.033). Coping strategy and positive emotion were positively correlated with IgG concentration (*P*=0.047 and *P*=0.008). Reward and sleep quality were positively correlated with IgM concentration (*P*=0.030, 0.000). Internal input was negatively correlated with IgM concentration (*P*=0.032). Reward and sleep quality were positively correlated with IgA concentration (*P*=0.047, 0.044). There were no significant correlations between other variables and glycosylated hemoglobin, triglyceride, total cholesterol and IgG, IgM, IgA levels (Table 3).

**Table 3:** Results of partial correlation analysis of occupational stress-related factors and glycosylated hemoglobin, triglyceride, total cholesterol and IgG, IgM, IgA levels

***Variable***	***Glycated hemoglobin***	***Triglyceride***	***Total cholesterol***	***IgG***	***IgM***	***IgA***
Effort	0.221	0.104	0.078	−0.003	0.113	0.029
Internal input	0.032	0.151^a^	0.063	0.057	−0.087^a^	−0.069
Reward	0.107	0.134	0.078	0.028	0.124^a^	0.077^a^
Positive emotion	0.061	0.003	0.044	0.114^b^	0.146	−0.069
Negative emotion	0.082^a^	−0.087	−0.004^a^	−0.009	−0.005	0.068
Coping strategy	−0.157 ^a^	0.049	0.055	0.095 ^a^	0.155	−0.083
Sleep quality	0.285	0.099	0.048	0.021	0.211^a^	0.982^a^

Notes: Relationships between occupational stress related factors and glycosylated hemoglobin, triglyceride, total cholesterol and IgG, IgM, IgA levels were analyzed by partial correlation analysis,

a *P*<0.05;

b *P*<0.01

### Covariance analysis between occupational stress and glycosylated hemoglobin, triglyceride, total cholesterol and IgG, IgM, IgA levels

There were significant differences in glycosylated hemoglobin, triglyceride, IgG, and IgM levels between high and low tension groups (*P*=0.030, *P*=0.009, *P*=0.035, *P*=0.045). Levels of glycated hemoglobin and triglyceride in high tension group were higher than those in low tension group. High tension group had lower IgG and IgM levels than low tension group. Differences in total cholesterol and IgA levels between the groups were not statistically significant (Table 4).

**Table 4:** Results of covariance analysis between occupational stress and glycated hemoglobin, triglycerides, total cholesterol, and IgG, IgM, and IgA levels

***Occupational stress***	***Glycated hemoglobin level (%)***	***Triglyceride (mmol/L)***	***Total cholesterol (mmol/L)***	***IgG (g/L)***	***IgM (g/L)***	***IgA (g/L)***
Low tension	5.10±0.32	1.49±0.79	4.88±0.71	11.43±3.04	1.21±0.40	2.38±0.59
High tension	5.16±0.21^a^	1.61±0.77^b^	4.86±0.75	11.37±3.01^a^	1.16±0.37^a^	2.40±0.57

Note: Relationships between occupational stress and glycosylated hemoglobin, triglyceride, total cholesterol and IgG, IgM, IgA levels were subjected to covariance analysis,

a *P*<0.05;

b *P*<0.01

### Logistic regression analysis with glycosylated hemoglobin, triglyceride, total cholesterol and IgG, IgM, IgA levels as dependent variables

In the model of glycosylated hemoglobin as the dependent variable, age and coping strategies entered the equation. In the model of triglyceride as a dependent variable, drinking and internal input entered the equation. In the model of total cholesterol as a dependent variable, drinking and negative emotions entered the equation. In the model of IgG concentration as a dependent variable, smoking, coping strategies, and positive emotions entered the equation. In the model of IgM concentration as a dependent variable, reward and quality of sleep entered the equation. In the model with IgA concentration as the dependent variable, only the quality of sleep entered the equation (Table 5).

**Table 5:** Statistically significant results in logistic regression analysis

***Dependent variable***	***Independent variable***	***Β***	***Standard error***	***Waldχ^2^***	***p***	***OR(95%CI)***
Glycated hemoglobin	Age					
	< = 35	-	-	3.225	-	1
	35∼50	0.224	0.312	3.228	0.075	0.857 (0.398∼0.947)
	>50	0.994	0.379	5.142	0.042	1.924 (1.001∼4.948)
	Coping strategy	−0.812	0.438	2.016	0.038	0.337 (0.027∼0.914)
Triglyceride	Drinking					
	No	-	-	2.785	-	1
	Yes	1.219	0.647	3.518	0.022	2.083 (1.901∼7.048)
	Internal input	1.022	0.515	4.017	0.047	1.212 (1.007∼4.141)
Total cholesterol	Drinking					
	No	-	-	1.648	-	1
	Yes	1.335	0.529	2.947	0.033	1.158 (1.002∼5.014)
	Negative emotion	1.041	0.418	3.074	0.032	1.504 (1.012∼3.365)
IgG	Smoking					
	No	-	-	4.227	-	1
	Yes	−0.867	0.328	6.142	0.012	0.428 (0.211∼0.917)
	Coping strategy	0.629	0.312	4.025	0.034	1.571 (1.201∼5.984)
	Positive emotion	0.885	0.427	3.583	0.040	2.493 (1.250∼6.907)
IgM	Reward	0.566	0.351	4.237	0.041	2.434 (1.212∼6.957)
	Sleep quality	0.624	0.347	4.112	0.036	1.582 (1.030∼4.988)
IgA	Sleep quality	0.882	0.532	6.174	0.021	1.527 (1.007∼6.897)

## Discussion

Cardiovascular diseases and metabolic diseases are common and multiple diseases that pose a serious threat to human health in modern society. With the development of the bio-psycho-social medical model, people gradually realize the importance of social psychological factors such as occupational stress in the occurrence, development, prevention and control of diseases. At the same time, the study of the impact of occupational stress on human immune function has gradually attracted people’s attention and developed into a new independent discipline-neuro-immunology or psycho-neuroimmunology.

Glycated hemoglobin, triglyceride and cholesterol are important risk factors for cardiovascular diseases and metabolic diseases, and are commonly used indicators for scholars to study the effects of occupational stress on the cardiovascular system, metabolic system or endocrine system. Coping strategies are related to low levels of glycated hemoglobin, and active coping strategies protect the metabolic system, that is, coping strategies help control blood sugar levels (10–12).

The present study found that occupational stress led to elevated levels of glycated hemoglobin. The higher the coping strategy, the lower the risk of elevated glycated hemoglobin levels. The effect of coping strategy on glycosylated hemoglobin levels was less than that of age. The conclusions of this study are consistent with the previous studies, indicating that coping strategies can reduce the risk of elevated glycated hemoglobin levels.

Negative emotions increase the risk of metabolic disease and are positively correlated with glycosylated hemoglobin levels (13). This study showed that there was a correlation between negative emotion and glycosylated hemoglobin level, but negative emotion did not enter the regression equation of glycosylated hemoglobin level as the dependent variable, suggesting that negative emotion has less influence on glycosylated hemoglobin level than coping strategy and age. The results of the study differ from those reported in previous studies, which may be explained by the differences research subjects and sentiment scales (13,14). A study with age, education, smoking, and alcohol consumption corrections (15) showed that occupational stress index was significantly associated with serum total cholesterol. However, this study found no significant difference in total cholesterol levels between the high and low tension groups.

However, regression analysis suggests that drinking and negative emotions are the key factors that increase the risk of elevated total cholesterol levels. Occupational stress can increase triglyceride levels (16). The results of this study indicate that occupational stress is associated with elevated triglyceride levels. The risk of triglyceride elevation in drinkers is 2.083 times that of non-drinkers, and the risk of elevated triglyceride levels in doctors with more internal inputs is 1.212 times that of less internal input doctors. This is consistent with another conclusion (17), and also in line with the physiological mechanism of occupational stress. In addition to providing more calories to the body, ethanol can also stimulate triglyceride synthesis and increase blood triglycerides. Among many occupational stress-related factors, internal input can lead to occupational stress, resulting in increased synthesis of catecholamines and increased levels of free fatty acids and glucagon in the blood (18).

Occupational stress has an inhibitory effect on the body’s humoral immune function, and the content of IgG and IgA decreases with the increase of occupational stress (6,19). However, the levels of IgG and IgA in the serum of highly stressed people are significantly higher than those in the control group, and occupational stress is positively correlated with the level of immune function (20). This study found that occupational stress has an effect on IgG and IgM levels. Among them, coping strategies, positive emotions and rewards can reduce the risk of decreased IgG and IgM concentrations, while good sleep quality can also reduce the risk of decreased IgM and IgA concentrations. In occupational stress studies, rewards and coping strategies are considered to be the mitigating factors of occupational stress, and positive emotions can be used as a favorable factor to alleviate occupational stress.

According to the theory of occupational stress and the study of stress-related factors, the results of this study suggest that occupational stress has an inhibitory effect on the body’s immune function, which is partially consistent with the results reported in previous literatures.

## Conclusion

Occupational stress can lead to increased blood lipids and sugar levels as well suppression of immune function in doctors.

## Ethical considerations

Ethical issues (Including plagiarism, informed consent, misconduct, data fabrication and/or falsification, double publication and/or submission, redundancy, etc.) have been completely observed by the authors.

## References

[1] Nyberg ST Heikkila K Fransson EI (2012). Job strain in relation to body mass index: pooled analysis of 160 000 adults from 13 cohort studies. J Intern Med, 272(1): 65–73. 22077620 10.1111/j.1365-2796.2011.02482.x PMC3437471

[2] Bergmann N Gyntelberg F Faber J (2014). The appraisal of chronic stress and the development of the metabolic syndrome: a systematic review of prospective cohort studies. Endocr Connect, 3(2): R55–80. 24743684 10.1530/EC-14-0031 PMC4025474

[3] Kivimaki M Singh-Manoux A Nyberg S Jokela M Virtanen M (2015). Job strain and risk of obesity: systematic review and meta-analysis of cohort studies. Int J Obes (Lond), 39(11): 1597–600. 26041697 10.1038/ijo.2015.103 PMC4579559

[4] Li C Xing JJ Shan AQ (2016). Increased risk of nonalcoholic fatty liver disease with occupational stress in Chinese policemen: A 4-year cohort study. Medicine (Baltimore), 95(46): e5359. 27861366 10.1097/MD.0000000000005359 PMC5120923

[5] Heraclides AM Chandola T Witte DR Brunner EJ (2012). Work stress, obesity and the risk of type 2 diabetes: gender-specific bidirectional effect in the Whitehall II study. Obesity (Silver Spring), 20(2): 428–33. 21593804 10.1038/oby.2011.95

[6] Nakata A Irie M Takahashi M (2013). A single-item global job satisfaction measure is associated with quantitative blood immune indices in white-collar employees. Ind Health, 51(2): 193–201. 23196390 10.2486/indhealth.2012-0059 PMC4683928

[7] Kumar S Sinha P Dutu G (2013). Being satisfied at work does affect burnout among psychiatrists: a national follow-up study from New Zealand. Int J Soc Psychiatry, 59(5): 460–7. 22518020 10.1177/0020764012440675

[8] Fahlen G Knutsson A Peter R Akerstedt T Nordin M Alfredsson L Westerholm P (2006). Effort-reward imbalance, sleep disturbances and fatigue. Int Arch Occup Environ Health, 79(5): 371–8. 16362323 10.1007/s00420-005-0063-6

[9] Yu SF Nakata A Gu GZ Swanson NG Zhou WH He LH Wang S (2013). Co-effect of Demand-control-support model and effort-reward imbalance model on depression risk estimation in humans: findings from Henan Province of China. Biomed Environ Sci, 26(12): 962–71. 24393505 10.3967/bes2013.031 PMC4701206

[10] Feldman PJ Steptoe A (2003). Psychosocial and socioeconomic factors associated with glycated hemoglobin in nondiabetic middle-aged men and women. Health Psychol, 22(4): 398–405. 12940396 10.1037/0278-6133.22.4.398

[11] Yancura LA Aldwin CM Levenson MR Spiro A 3rd (2006). Coping, affect, and the metabolic syndrome in older men: how does coping get under the skin? J Gerontol B Psychol Sci Soc Sci, 61(5): P295–303. 16960233 10.1093/geronb/61.5.p295

[12] Tsenkova VK Dienberg Love G Singer BH Ryff CD (2008). Coping and positive affect predict longitudinal change in glycosylated hemoglobin. Health Psychol, 27(2S): S163–71. 18377158 10.1037/0278-6133.27.2(Suppl.).S163

[13] Ciechanowski PS Katon WJ Russo JE Hirsch IB (2003). The relationship of depressive symptoms to symptom reporting, self-care and glucose control in diabetes. Gen Hosp Psychiatry, 25(4): 246–52. 12850656 10.1016/s0163-8343(03)00055-0

[14] Nyberg ST Fransson EI Heikkila K (2014). Job strain as a risk factor for type 2 diabetes: a pooled analysis of 124,808 men and women. Diabetes Care, 37(8): 2268–75. 25061139 10.2337/dc13-2936 PMC4113178

[15] Su CT Yang HJ Lin CF Tsai MC Shieh YH Chiu WT (2001). Arterial blood pressure and blood lipids as cardiovascular risk factors and occupational stress in Taiwan. Int J Cardiol, 81(2–3): 181–7. 11744135 10.1016/s0167-5273(01)00565-4

[16] Belkic KL Landsbergis PA Schnall PL Baker D (2004). Is job strain a major source of cardiovascular disease risk? Scand J Work Environ Health, 30(2): 85–128. 15127782 10.5271/sjweh.769

[17] Kobayashi Y Hirose T Tada Y Tsutsumi A Kawakami N (2005). Relationship between two job stress models and coronary risk factors among Japanese part-time female employees of a retail company. J Occup Health, 47(3): 201–10. 15953841 10.1539/joh.47.201

[18] Salemi G Gueli MC Vitale F (2010). Blood lipids, homocysteine, stress factors, and vitamins in clinically stable multiple sclerosis patients. Lipids Health Dis, 9: 19. 20163740 10.1186/1476-511X-9-19 PMC2834681

[19] Nakata A Takahashi M Irie M (2011). Effort-reward imbalance, overcommitment, and cellular immune measures among white-collar employees. Biol Psychol, 88(2–3): 270–9. 21889570 10.1016/j.biopsycho.2011.08.012

[20] Marotta F Naito Y Padrini F (2011). Redox balance signalling in occupational stress: modification by nutraceutical intervention. J Biol Regul Homeost Agents, 25(2): 221–9. 21880211

